# Effect of gold nanoparticles treatment on rats-induced obesity by evaluating body-composition directly and indirectly via bioelectric impedance analysis

**DOI:** 10.1038/s41598-025-87971-z

**Published:** 2025-02-10

**Authors:** Rana M. Selima, Israa A. Saleem, Mamdouh M. Shawki, Amira A. Darwish, Mona A. Yehia, Ehab I. Mohamed

**Affiliations:** 1 Medical Biophysics Department, Medical Research Institute, Alexandria University, 165 El-Horreya Avenue, Alexandria, 5433005 Egypt; 2 Optometry Department, Technical Medical Institute, Erbil Polytechnic University, Erbil, Iraq; 3 Medical Laboratory Technology Department, Faculty of Applied Health Sciences Technology, Pharos University, Alexandria, Egypt; 4 Histochemistry and Cell Biology Department, Medical Research Institute, Alexandria University, Alexandria, Egypt

**Keywords:** Obesity, Gold nanoparticles (AuNPs), Body-composition, Bioelectric Impedance Analysis (BIA), Dyslipidemias, Oxidative damage, Biophysics, Drug discovery, Health care, Medical research

## Abstract

Obesity is a metabolic disease characterized by an imbalance between caloric intake and expenditure, leading to excess fat and increasing the risk of various health conditions. This study compares the anti-obesity effects of gold nanoparticles (AuNPs) to orlistat in an experimental model of induced obesity in Wistar Albino rats. In addition to negative and positive control rats, obese rats were treated with variable daily and weekly doses of AuNPs and daily orlistat for nine weeks. Bioelectric impedance analysis (BIA) and dissection techniques were used to indirectly and directly measure body-composition in all rat groups. Hepatic and renal function and ultrastructure were assessed by blood biochemical and histological examinations to detect treatment-related alterations. High doses of AuNPs reduced body fat, increased muscle mass, improved dyslipidemia, glycemia, and antioxidant effects in obese rats, and restored normal TG, FBG, and MDA levels by reducing obesity-related oxidative damage. Histological and ultrastructural examinations showed that these high doses repaired liver and kidney cells, and reduced fat accumulation and body weight compared to the standard treatment for obesity by orlistat.

## Introduction

Obesity is a multifactorial metabolic disease characterized by an imbalance between caloric intake and expenditure, which manifests as an excess of fat in tissues and a body mass index (BMI) of ≥ 30 kg/m^2^^1,2^. Obesity increases the risk of a multitude of health conditions, including hyperglycemia, type II diabetes mellitus, hypertension, coronary heart disease, osteoarthritis, gallstones, non-alcoholic fatty liver, sleep apnea, and many cancers^1,3,4^. With 1.9 billion overweight adults and 650 million obese individuals, the prevalence of obesity tripled globally between 1975 and 2016, according to the WHO, highlighting the growing epidemic of obesity as a global concern^5^. Egypt has the 18th highest obesity prevalence globally, with adult obesity rates at 39.8%, linked to increased comorbidities and mortality rates, according to the 2019 Egyptian “100 million health” survey. The Egyptian economy loses around LE 62 billion yearly due to obesity, which causes 4 million disability-adjusted life years and 115,000 deaths per year (19.08% of the overall deaths in 2020)^6^. Therefore, since an estimated 60% of the world’s population may be overweight or obese by 2030, we urgently need innovative therapies for obesity^7^.

The limited effectiveness of currently available drugs, such as orlistat, in managing human obesity is due to their serious side effects and poor efficacy^7^. Given their non-immunogenicity, hydrophilicity, biocompatibility, and higher cell uptake, researchers are investigating surface-modified gold nanoparticles (AuNPs) for potential use in biomedicine^7,8^. Studies have demonstrated the effectiveness of AuNPs in preventing weight gain, reducing fat deposition, and reducing metabolic inflammation and endotoxemia in mice with high-fat diet (HFD)-induced obesity^9^. It has also been shown that intraperitoneal administration of non-modified AuNPs could significantly reduce abdominal fat tissue and inhibit inflammatory effects in mice without causing organ or cell toxicity^9–11^. AuNPs accumulation in abdominal visceral fat might lower levels of tumor necrosis factor-alpha (TNF-α), a cytokine that causes inflammation and is strongly linked to insulin resistance and a number of metabolic disorders^12^.

Calculated as the ratio of a person’s weight (kg) to their square height (m^2^), the body mass index (BMI) is not a reliable indicator of health because it cannot reveal details about a person’s fat content or any other body component^13^. Bioelectrical impedance analysis (BIA) measures the electrical response of the body and is thus an indirect method that may analyze body-composition; as a result, it is a reliable indicator of obesity and overall health^14^. BIA in biological cells is influenced by the frequency of alternating current inducted into the body and the composition of biological tissues. Intra- and extracellular fluids, which are the main parts of body tissues, function as resistors due to their electrical conductivity, while cell membranes, composed of non-conducting lipids sandwiched between two conducting protein layers, function as capacitors^15^. This non-invasive technique could be used to assess nutrition status and estimate body-composition [i.e., fat-free mass (FFM), skeletal muscle mass (SMM), fat mass (FM), and total body water (TBW)] rapidly and precisely^15–17^. In an early study on HFD-induced obesity rats, TBW and FFM predictions made using BIA were, on average, within 5% of the values determined using the tritiated water^3^H_2_O) dilution technique^18^. Further, the BIA technique showed a BMI-dependent decrease in phase angle values of Italian patients ranging from mild to severe obesity, emphasizing its potential for assessing body-composition specific to obesity^19^.

This study is aimed at assessing the impact of AuNPs administration on the health status and body-composition in an HFD-induced obesity rat experimental model.

## Materials and methods

### AuNPs characterization

Aqueous suspension of citrate-capped AuNPs was obtained from Nawah Scientific in Cairo, Egypt. The concentration of the suspension was 200 ppm, and the size of the AuNPs was 21 ± 5 nm, according to the manufacturer. The absorbance of AuNPs solutions was measured throughout a wavelength range of 200 to 800 nm using an ultraviolet-visible (UV-Vis) double-beam spectrophotometer (Helios Alpha 9423 UVA 1002E UVVis, Thermo Spectronic, UK). The crystallite sizes and phases of AuNPs were examined using a powder X-ray diffractometer (Discover D8–XRD, Bruker, USA) configured with a CuK target at a λ of 1.5406 Å, a voltage of 40 kV, and a current of 30 mA. A range of 2θ from 5° to 100°, with a step size of 0.05°, was used to examine the X-ray diffraction (XRD) patterns of AuNPs samples. Moreover, the composition and functional groups of aqueous AuNPs were investigated in the range between 400 and 4000 cm^− 1^ using a Fourier transform of infrared (FTIR) spectrometer (Nicolet iS50 FTIR Spectrometer, ThermoFisher Scientific Inc., USA) equipped with a deuterated L-alanine-doped triglycine sulfate (DLaTGS) detector.

Furthermore, the size, shape, and surface morphology of AuNPs were examined under a scanning electron microscope (SEM) (JSM-IT200, JEOL, Japan). The sample was placed on a glass slide, attached to the SEM stub using carbon double-face adhesive tape, allowed to dry for several hours, coated with Au, and imaged at a magnification of 30,000×. Finally, the stability and aggregation of AuNPs were evaluated at room temperature by measuring their molecular sizes, concentrations, and surface charges using a zeta potential (ZP) analyzer (Zetasizer, Zen 3600, Malvern Panalytical, UK).

### AuNPs cytotoxicin vitro effects on human skin fibroblast (HSF)

Cultures of human skin fibroblast (HSF) (Innoprot, Derio–Bizkaia, Spain) cells were grown at 37 °C in a humidified atmosphere of 5% (v/v) CO_2_, in a medium that contained 10% heat-inactivated fetal bovine serum, penicillin (100 u/ml), and streptomycin (100 mg/ml). A 96-well plate was filled with 100 µl of cell suspension (5 × 10^3^ cells) and left to incubate in full medium for 24 h. UV-sterilized cells were then treated for 72 h with five different concentrations of AuNPs (i.e., 0.005, 0.05, 0.5, 5, and 50 µg/ml); thereafter, they were compared to untreated cells. There was 150 µl of 10% trichloroacetic acid used to fix the grown HSF cells for an hour at 4 °C. The cells were then washed five times with distilled water and stained with 70 µl of a 0.4% w/v Sulforhodamine B (SRB) solution. The plates were incubated in a dark place for 10 min. at room temperature, washed three times with 1% acetic acid, and air-dried overnight. Protein-bound SRB stain was dissolved using TRIS (10 mM; 150 µl). Finally, the absorbance was measured at 540 nm using an ELISA microplate reader (FLUOstar Omega, BMG LABTECH^®^, Ortenberg, Germany). Treated and untreated cells were examined under a light microscope, and high-resolution images were taken using a digital camera at the same magnification of 100×^20,21^.

### Obesity in vivo examination

#### Experimental design

At the beginning of this nine-week experiment, a total of 48 adult male Wistar Albino rats (Charles River Laboratories, Wilmington, MA, USA) of about two months old were divided into six equal groups (*n* = 8). Eight rats received a standard commercial rat chow as a negative control group, G-I. In addition, the other 40 rats were given an HFD to induce obesity for 9 weeks. They were divided into five groups: G-II, the positive control group, which received no treatment; G-III, the accumulated high-dose group, which received a daily *i.p.* injection of AuNPs at 7.48 µg/kg; G-IV, the weekly high-dose group, which received 52.33 µg/kg; G-V, the weekly low-dose group, which received 5.23 µg/kg; and G-VI, the orlistat group, which received a daily orlistat *i.p.* injection at a dose of 12 mg/kg dissolved in 1 ml/kg saline.

### Animals

A total of 48 male Wistar Albino rats, 50–60 days old, with a weight range of 150–160 g at the beginning of the experiment, were randomly divided into six equal groups (*n* = 8). Rats were acclimatized for a couple of weeks in air-conditioned rooms at a temperature of 25 °C and a 12-hour light/dark cycle at the animal house of El-Mowasah Educational and Medical Complex, Alexandria University, Egypt. The rats were housed in spacious, rectangular polypropylene cages that had steel-nozzle water bottles and stainless-steel covers. Up to four adult rats could fit in each cage, and they received free drinking water and food according to the experimental design.

Prior to the beginning of experiments, the study protocol received approval from the Institutional Animal Care and Use Committee of Alexandria University, with the reference number IACUC # 01221112411. Subsequently, the study protocol received definitive clearance from the Ethics Committee of the Medical Research Institute at Alexandria University in Alexandria, Egypt. All the methods employed and detailed in this study adhered to the criteria established by the ARRIVE guidelines. Furthermore, the study followed the US National Institutes of Health’s Guide for the Care and Use of Laboratory Animals (NIH publication no. 85 − 23, revised 1996)^22^.

### Induction of obesity

This study examined a Wistar Albino rat model with diet-induced obesity to investigate the effects of obesity and its associated consequences. Rat groups G-II to G-VI were fed an HFD with 58% fat, 11% carbohydrates, 21% protein, and 2.8% crude fiber for 9 weeks. Rat group G-I, the negative control group, received a standard commercial rat chow containing 14% protein.

### Blood sampling and biochemical parameters

Blood samples were collected from 12-hour overnight fasted rats that had been anesthetized with an *i.m.* injection of ketamine (10 mg/kg) and xylazine (15 mg/kg)^23^. The retro-orbital bleeding technique was used by penetrating the retro-orbital plexus with a sterile hematocrit capillary tube (length: 75 mm, inner diameter: 1.1 mm, and outer diameter: 1.5 mm) to provide blood samples of adequate volume^24^. All samples were then centrifuged at 4000 rpm for 10 min, and the supernatant was pipetted and stored at -20 °C for evaluation of blood biochemical parameters. Standard chemical and enzymatic assays were used to measure serum concentrations of total cholesterol (TC), low-density lipoprotein cholesterol (LDL-C), high-density lipoprotein cholesterol (HDL-C), triglycerides (TG), fasting blood glucose (FBG), and malondialdehyde (MDA)^25–27^.

### Biometric measurements

Rats’ body weight (BW) was recorded once weekly using a digital precision balance scale (PNX-602, American Weigh Scales, USA) with a 600 g capacity and a 0.1 g precision. The rat’s body length was measured while the rat was lying on its abdomen using a ruler starting from the nasal bone to the anus. The body mass index (BMI, g/cm^2^) was calculated by dividing the weight (g) by the squared body length (cm^2^) of each rat. Moreover, the Lee index (LI, g^1/3^/cm) was calculated as the cube root of weight (g^1/3^) divided by the naso-anal length (cm) of each rat and multiplied by 1000^28^.

### Indirect body-composition analysis using BIA technique

**Fig. 1:**
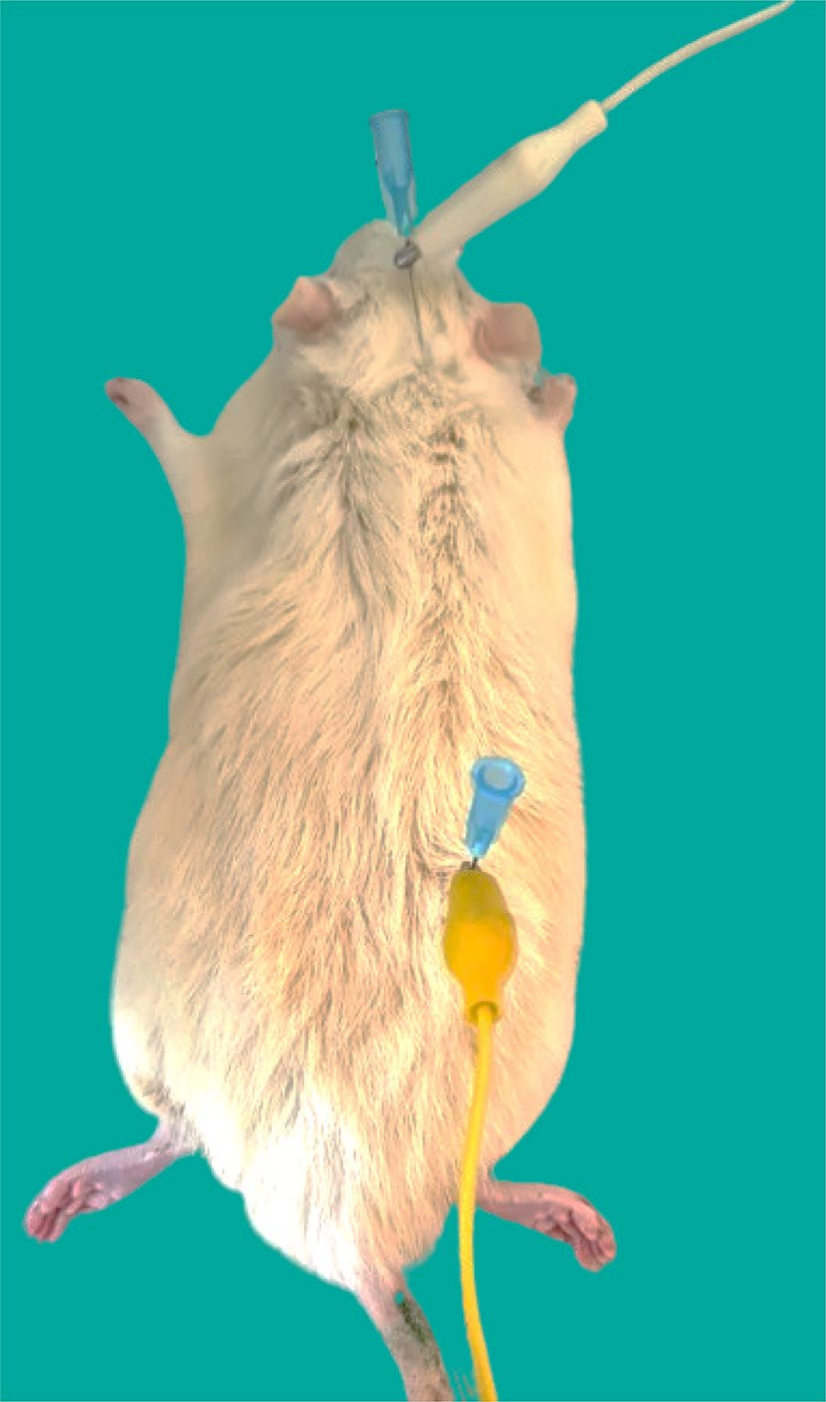
The two needle electrodes setup for measuring a rat’s body-composition, with one needle placed between the ears and the other near the tail’s base, where the thigh muscles meet, across the dorsal median line.

The BIA technique was used to study the rat’s body-composition, where the whole-body impedance was measured using a two-electrode technique of an LCR meter (Hioki 3532-50 LCR Hitester) at 50 kHz, as detailed elsewhere^18,29^. Two 23G stainless-steel needles (32 × 0.6 mm) were inserted subcutaneously (5–10 mm deep) along the dorsal area of an anesthetized rat lying on its abdomen, with its four limbs alongside the trunk and tail extended. As shown in Fig. 1, the two needles were placed across a rat’s dorsal median line, one between its ears and the other near the base of its tail, where the muscles of its thighs meet. One of these needles functioned as an electrode, inducing a low-intensity AC current through the rat’s tissues; the other detected the potential and the animal’s whole-body impedance^30^.

The distance between the electrodes was measured, and the electrical parameters, such as resistance and capacitance, were observed on the monitor of an LCR meter. Then, parameters like fat-free mass (FFM), fat mass (FM), fat percentage (Fat %), and total body water (TBW) were calculated using the formulae given by Eq. (1) to Eq. (5):^18^.

1 $$\text{FFM}=0.38 \times \text{BW}+13.8 \times \:\frac{{L}^{2}}{{R}_{50}}+70.9$$

2 $$\text{FM}=\text{BW}-\text{FFM}$$

3 $$\text{Fat} \% = \:\frac{\text{F}\text{M}}{\text{B}\text{W}} \times 100$$

4 $${\text{TBW}} = 309.9 \times \frac{{{L^2}}}{{{Z_c}}} + 30.0$$

Where *L* is the length between the two needle electrodes (cm), BW is the rat’s body weight (g), *R*_50_ is the resistance (ohm) at a frequency of 50 kHz, and *Z*_*c*_ is the impedance (ohm), which is given by Eq. (5):^29^.

5 $$\:{Z}_{C}\:=\:\sqrt{{R}^{2} + {{X}_{c}}^{2}}$$

Where *X*_*c*_ is capacitive reactance (ohm), which is determined by Eq. (6):^31^.

6 $$\:{X}_{c}=\:\frac{1}{2\pi\:fC}$$

Where *C* is the dielectric capacitance (pF) and *f* is the frequency that is equal to 50 kHz.

### Direct body-composition analysis

By the end of the 9th week, anesthetized rats were euthanized by cervical dislocation, a method providing a fast, painless, and easy death for the animal^32^. Subsequently, the rat’s fur, skin, tail, feet, and visceral organs were dissected, allowing for a direct evaluation of its main body-composition from carcasses of bones, muscles, and fat. An analytical balance (AS 220.R2, Radwag, Poland) was used to weigh the bones, muscles, and subcutaneous and visceral fat. Bones and muscles were then dried in an oven (Blue M, Stabil-Therm Gravity Oven, New Columbia, PA, USA) at 105 °C for three hrs to calculate their water content by subtracting the dry weight of the tissues from their initial weight^33^.

### Histopathological analysis

Kidneys and livers were studied histopathologically using light and transmission electron microscopy (TEM) in negative control and obese rat groups. Specimens from both organs were sliced and fixed in 10% formalin, dehydrated in ascending concentrations of alcohol from 70 to 100%, and cleared using xylene. Subsequently, specimens were submerged in molten paraffin wax at 60 °C for 1–2 h. After the paraffin cooled and hardened into blocks, the tissues were sectioned into 5 μm-thick slices with a microtome. These slices were then put on clean glass slides and stained with hematoxylin and eosin (H&E)^34^. The slides were finally examined, and 400× magnification photographs were captured using a light microscope equipped with a 14.0 MP HD digital camera (Model BX41, Olympus, Japan).

Further specimens of liver and kidney were fixed with 4F1G in a phosphate buffer solution (pH 7.2–7.4) at 4 °C for 1 h and then fixed again for 2 h at 4 °C in a 2% OsO_4_-phosphate buffer solution. The specimens were dehydrated using a series of acetone gradients at 4 °C. They were then embedded in resin, cut into 90 Å sections with an ultra-microtome, and mounted on TEM grids. The sections were stained with uranyl acetate for 5 min and lead citrate for 2 min before being analyzed at magnifications ranging from 1200× to 15,000× using a TEM (JSM-1400PLUS, JEOL, Japan).

### Blinding and masking

The six researchers employed the following method to analyze each animal: Two researchers, RMS and IAS, used the experimental design to randomly assign rats to groups and then treat obese rats with AuNPs and orlistat therapy. Only these two researchers were privy to the treatment protocols for the rats. AAD, a third researcher, oversaw both anesthesia and euthanasia. Finally, three researchers (MMS, MAY, EIM) were blinded to the obesity therapies and evaluated the body-composition, biochemical, and histopathological outcomes in rats.

### Outcome measurements

Depending on the concentration of AuNPs used to treat obesity, rats with the condition showed varying degrees of improvement after 9 weeks. These included lower levels of BMI, LI, FM, FP, HDL-C, TG, FBG, and MDA, as well as higher levels of FFM, TBW, TC, and LDL-C as assessed by body-composition, biochemical, and histopathological analysis.

### Statistical analysis

The statistical package SPSS (Version 16; Chicago, IL, USA) was used for the quantitative analysis of the data. To compare normally distributed variables across groups, the analysis of variance (ANOVA) test was applied, and to compare them between pairs, the post hoc test (Tukey) was employed. The results were displayed as the mean plus or minus the standard deviation (± SD), with a *p*-value less than 0.05 indicating statistical significance.

## Results and discussion

Obesity and dyslipidemia are mostly manifested by the malfunction in adipose tissue and are interrelated through analogous pathways and therapies, which are ineffective, non-specific, and cause harmful side effects^35^. Since targeted nanotherapy has high tolerability, tailored cellular delivery, degradation prevention, longer drug release, and minimized adverse effects, it may be an innovative treatment for both disorders. Sibuyi et al.,^36^ initially suggested nanotechnology-based therapies for treating obesity by inhibiting the processes of white adipose tissue angiogenesis, brown adipose tissue transformation, and photothermal lipolysis. Here we detail a clinical trial in animals that compares the efficacy of various concentrations of AuNPs for the treatment of obesity to that of conventional orlistat medications.

### AuNPs characterization

**Fig. 2:**
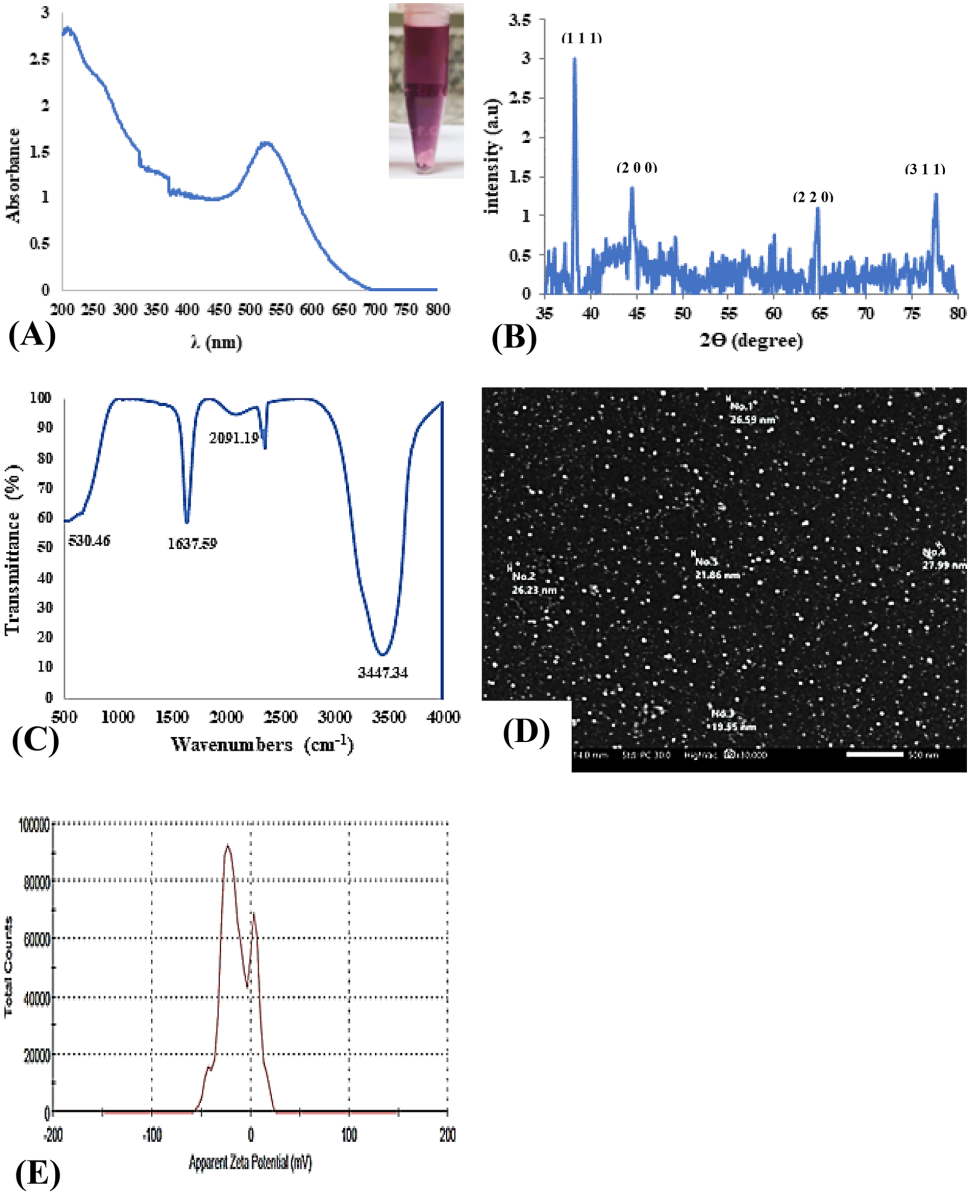
Characterization measurements of aqueous gold nanoparticles (AuNPs) showing their absorbance values of ultraviolet-visible spectra (**A**), X-ray diffraction (XRD) patterns (**B**), Fourier transform of infrared (FTIR) spectra (**C**), scanning electron microscope (SEM) at a magnification of 30,000× (**D**), and Zeta-potential distribution (**E**).

The UV-Vis absorbance spectra of citrate-capped AuNPs, which were red wine-colored suspensions in water, showed a distinct peak at 531 nm (Fig. 2A). This color and AuNPs’ characteristic absorbance peak, which was attributed to surface plasmon resonance, agree with earlier findings^37,38^. Figure 2B shows the AuNPs crystallinity, unit cell dimensions, and lattice parameters after an XRD analysis, identifying four diffraction peaks at 2θ values of 38.31°, 44.46°, 64.67°, and 77.65°. These values correspond to (111), (200), (220), and (311) planes in a face-centered cubic lattice, respectively. Additionally, the observation of a sharp peak at 38.3° suggested that the (111) direction was ideal for the formation of AuNPs crystals^39,40^.

Figure 2C shows the functional groups of aqueous AuNPs identified by FTIR spectrometry, which contain a broad peak at 3447.34 cm^− 1^, a sharp peak at 1637.59 cm^− 1^, another at 530.47 cm^− 1^, and the weakest band at 2091.19 cm^− 1^. It has been previously revealed that the broad peak at 3447.34 cm^− 1^ represents the stretching vibration of the O-H group due to the strong water-hydrogen bond^41^. The peak at 1637.59 cm^− 1^ corresponds to carboxylate (COO^−^) asymmetric stretching vibration, while the peak at 530.47 cm^− 1^ is attributed to C-Cl stretching vibration^42^. The band at 2091.19 cm^− 1^ has been associated with the aromatic compounds on AuNPs’ surfaces, indicating the presence of a C-H group^43,44^.

Figure 2D displays the size, shape, and surface morphology of AuNPs as determined by SEM examination. The particles ranged in size from 18 to 30 nm, and their shape was spherical except for a few clusters of irregular shapes, which agree with recent findings^38,45^. As seen in Fig. 2E and also reported by Oliveira et al.,^38^ the average ZP of citrate-capped aqueous AuNPs had a distinct peak at -19.9 mV, which falls within the stable range of -30 to + 30 mV. Chemically synthesized AuNPs have been shown to be electrostatically repulsive and had a ZP of about −18.2 ± 1.1 mV, according to Jafarizad et al.^46^ Moreover, the negatively charged surfaces of AuNPs manufactured using stabilizing chemicals (e.g., citrate capping) prevent nanoparticles from aggregating due to electrostatic repulsiveness^47^.

### AuNPs cytotoxicin vitro effects on HSF

**Fig. 3:**
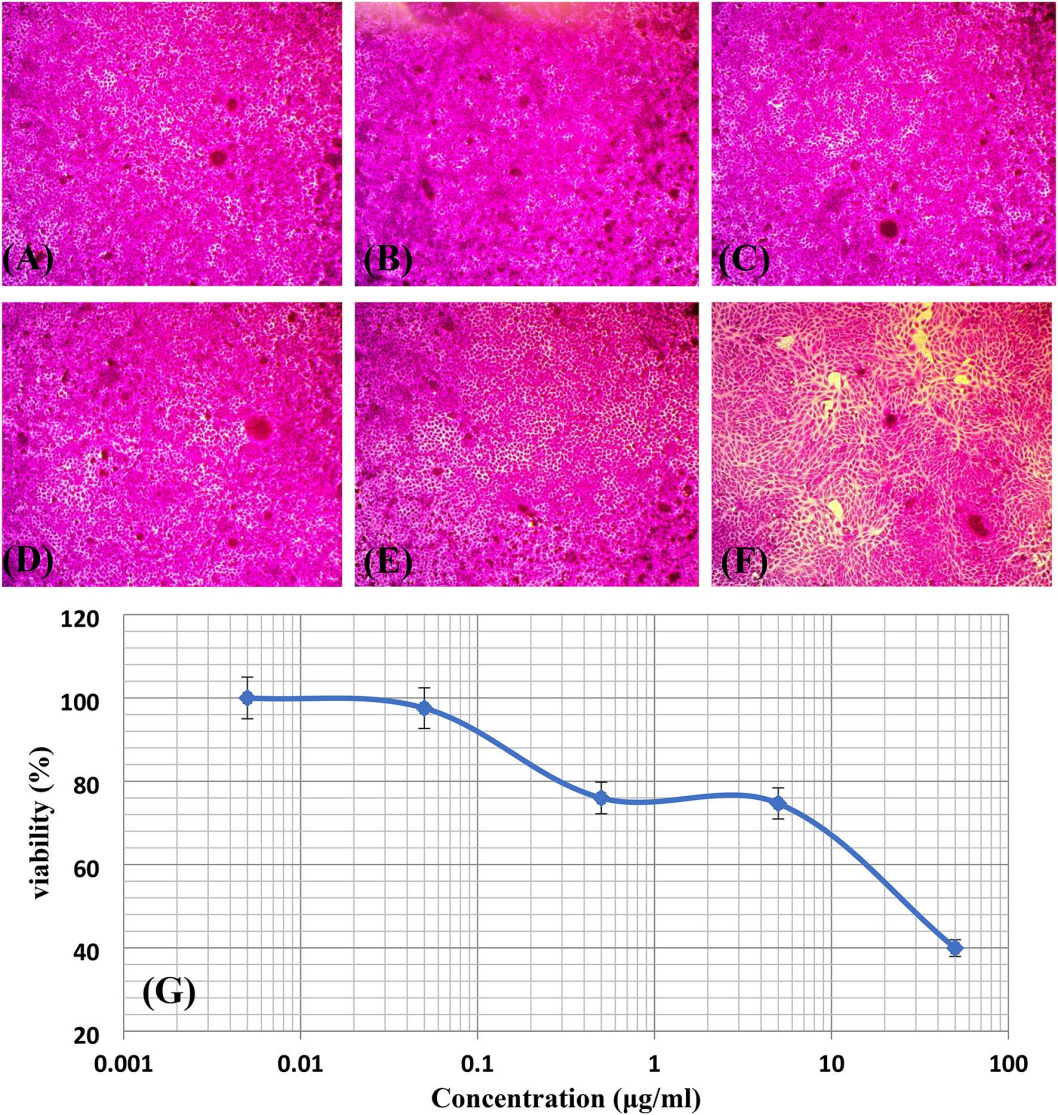
Images of the human skin fibroblast (HSF) cells’ viability as measured by the Sulforhodamine B (SRB) assay, captured under a light microscope at a magnification of 100×, showing controls (**A**) and cells at AuNPs concentrations of 0.005 (**B**), 0.05 (**C**), 0.5 (**D**), 5, and 50 µg/ml (**F**), along with a graphical representation of the viability percentages (**G**).

The results of HSF cells’ viability treated with different concentrations of AuNPs from 0.005 to 50 µg/ml are shown in Fig. 3A to F. The cell viability showed a concentration-dependent relationship (Fig. 3G), with HSF cells exposed to 0.005 and 0.05 µg/ml of AuNPs exhibiting no or negligible mortality, and 75.99 ± 1.1, 74.68 ± 2.2, and 39.94 ± 1.4% at concentrations of 0.5, 5, and 50 µg/ml of AuNPs, respectively. The concentration that inhibited 50% of cell viability was 25.47 µg/ml. Mateo et al.,^48^ have shown previously that the viability of normal HSFs treated with water-soluble AuNPs varied with dose, as measured by the MTT assay. Various diameters of citrate-capped AuNPs exhibited size-dependent cytotoxicity towards human erythrocytes and murine fibroblasts; the least toxic being 20 nm AuNPs, and the most harmful being 30 nm AuNPs^49^. The IC50 value of citrate-capped AuNPs for the noncancerous BHK cell line was 45 µg/ml^47^, yet each cell line reacts differently to the same nanoparticles under similar conditions^50^.

### Biometric measurements

This study investigated the effects of HFD-induced obesity in a Wistar Albino rat model, which accurately mimics human overweight and obesity^51^, to study obesity and metabolic disorders and gain insights into weight gain, body-composition, and metabolic parameters. Table 1 summarizes the biometric parameters (i.e., initial BW, final BW, weight gain, BMI, and LI) for all rat groups. The positive control obese rats G-II, as shown by an average LI of 343.40 ± 5.95 g^1/3^/cm, had a final BW that was more than twice as large as their initial BW compared to negative control rats G-I. In animal models, specifically rats, LI values exceeding 310 g^1/3^/cm indicate obesity^28^. After nine weeks of HFD, their weight gain, BMI, and LI were significantly (*p* < 0.05) higher than all the other groups. However, daily and weekly high doses of AuNPs (i.e., G-III: 7.48 and G-IV: 52.33 µg/kg) led to a significant (*p* < 0.05) weight loss in rats’ and consequently lower BMI and LI values compared to G-II. Low doses of AuNPs (G-V: 5.23 µg/kg) or orlistat (G-VI: 12 mg/kg) also significantly (*p* < 0.05) reduced the weight gain, BMI, and LI but to a lesser extent compared to AuNPs at higher doses (G-III and G-VI).

**Table 1:** Comparison of biometric parameters among all rat groups following treatment with varying dosages of AuNPs and orlistat.

	Initial BW (g)	Final BW (g)	Weight Gain (g)	BMI (g/cm^2^)	LI (g^1/3^/cm)
Negative controls (G-I)	162 ± 4.93	223 ± 5.29^bcdef^	61 ± 3.79^bcdef^	0.56 ± 0.01^bcdef^	303.19 ± 2.41^bcdef^
Positive controls (G-II)	150 ± 8.79	324 ± 16.72^acdef^	174 ± 10.77^acdef^	0.81 ± 0.04^acdef^	343.40 ± 5.95^acdef^
Accumulated high-dose (G-III)	157 ± 6.22	270 ± 15.06^abef^	113 ± 14.07^abef^	0.68 ± 0.04^abef^	323.18 ± 6.11^abef^
Weekly high-dose (G-IV)	157 ± 5.85	266 ± 15.33^abef^	109 ± 9.78^abef^	0.66 ± 0.04^abef^	321.27 ± 6.24^abef^
Weekly low-dose (G-V)	157 ± 5.62	293 ± 8.06^abcd^	136 ± 5.91^abcd^	0.73 ± 0.02^abcd^	331.88 ± 3.05^abcd^
Orlistat (G-VI)	155 ± 5.03	296 ± 8.50^abcd^	141 ± 3.61^abcd^	0.74 ± 0.02^abcd^	333.33 ± 3.19^abcd^

BW: Body weight; BMI: Body mass index; LI: Lee index.

Values are presented as mean ± SD. *P*-value $\:<$ 0.05 against: ^a^G-I, ^b^G-II, ^c^G-III, ^d^G-IV, ^e^G-V, and ^f^G-VI.

These results are in line with those of Chen et al.,^9^ who showed that obese mice had substantial weight loss after receiving daily *i.p.* injections of AuNPs (average diameter: 21 nm) for nine weeks. Green-synthesized AuNPs at a dose of 50 mg/kg showed a positive effect in managing obesity-related excessive weight and BMI in diabetic obese rats^52^. Prá et al.^53^. , found that AuNPs-treated rats (daily *i.p.* injection of 70 mg/kg for 14 days) had reduced dietary intake, inflammation, and oxidative stress but not BW or visceral fat weight. While earlier studies have shown that orlistat can reduce obesity-related weight gain^54,55^, AuNPs are a more effective anti-obesity treatment due to their anti-inflammatory and antioxidant effects, as well as their ability to accumulate in adipose tissues^53^.

Biometric measurements have been widely used to detect overweight and obesity, but they were considered inadequate since they could not measure the FM or FFM components^56^. Excess FM, particularly in visceral adipose tissues, was associated with hypertension, cardiovascular disease, type II diabetes, and a variety of other metabolic disorders^57^. Therefore, the BIA technique is gaining prominence as a noninvasive means of monitoring health and disease status, particularly in clinical trials^13,58^. It provides reliable estimates of fluid distribution and body-composition, is quick, portable, easy to use, and inexpensive^59^. Despite controversies, the BIA technique can give reliable estimates of FFM and TBW due to the resistance of fluids in bodily tissues (Eqs. 1 and 4), and it outperforms other methods for calculating FM from BW and FFM (Eq. 2)^14,60^.

### Indirect body-composition analysis

Table 2 summarizes the body-composition parameters (FM, FP, FFM, FM/FFM, TBW, and TBWP), evaluated indirectly using the BIA technique, for all rat groups. G-II rats had a significantly higher (*p* < 0.05) average FM, FP, FFM, and FM/FFM but lower TBW and TBWP after nine weeks of HFD. Although the G-II FFM values were higher compared to negative controls and all treated groups, obesity severely impacted the rats’ average muscle content, as evidenced by a higher FM/FFM ratio and lower TBW. Since muscle tissues contain a substantial amount of water (about 73% by weight), TBW measurements represent muscle mass^59,61^. Obese children and young adults aged 3–21 showed a significantly lower average TBW at weight and body surface area than normal weight individuals (i.e., 24.93 ± 0.37 vs. 26.94 ± 0.29 L, *p* < 0.001)^62^.

**Table 2:** Indirect body-composition analysis using the bioelectric impedance analysis (BIA) method for all study rat groups.

	FM (g)	FP (%)	FFM (g)	FM/FFM	TBW (g)	TBWP (%)
Negative controls (G-I)	64.87 ± 0.67^bcdef^	28.70 ± 0.00^bcdef^	161.13 ± 0.75^bcdef^	0.40 ± 0.00^bcdef^	127.48 ± 2.13^bcdef^	79.11 ± 0.01^bcdef^
Positive controls (G-II)	135.76 ± 2.72^acdef^	41.49 ± 0.02^acdef^	203.74 ± 1.26^acdef^	0.67 ± 0.01^acdef^	112.78 ± 0.97^acdef^	55.36 ± 0.00^acdef^
Accumulated high-dose (G-III)	90.81 ± 4.29^abef^	33.63 ± 0.01^abef^	174.86 ± 1.57^abef^	0.52 ± 0.02^abef^	119.96 ± 0.55^abef^	68.61 ± 0.01^abef^
Weekly high-dose (G-IV)	86.94 ± 4.81^abef^	32.68 ± 0.01^abef^	172.72 ± 2.59^abef^	0.50 ± 0.02^abef^	120.33 ± 0.31^abef^	69.67 ± 0.01^abef^
Weekly low-dose (G-V)	102.99 ± 4.81^abcd^	36.07 ± 0.01^abcd^	182.98 ± 3.12^abcd^	0.56 ± 0.02^abcd^	115.79 ± 0.62^abcd^	63.29 ± 0.01^abcd^
Orlistat (G-VI)	111.13 ± 2.67^abcd^	36.52 ± 0.01^abcd^	189.55 ± 2.61^abcd^	0.59 ± 0.01^abcd^	115.95 ± 0.76^abcd^	61.18 ± 0.00^abcd^

FM: Fat mass; FP: Fat percentage; FFM: Fat-free mass; TBW: Total body water; TBWP: Total body water percentage (i.e., TBW/FFM).

Values are presented as mean ± SD. *P*-value $\:<$ 0.05 against: ^a^G-I, ^b^G-II, ^c^G-III, ^d^G-IV, ^e^G-V, and ^f^G-VI.

High-dose treatment with AuNPs of G-III and G-IV significantly (*p* < 0.05) reduced FM and FP and restored the muscle contents by elevating TBW and lowering FM/FFM compared to non-treated obese rats G-II. Low doses of AuNPs and orlistat were comparable but had less pronounced FM, FP, and FM/FFM reductions and TBW elevation. Recent studies have associated excessive FM and higher FM/FMM ratios with metabolic disorders, chronic inflammation, non-alcoholic fatty liver disease, and cardiac abnormalities^63–66^. Thus, lowering FM and FM/FFM and increasing TBW show that AuNPs are better than orlistat for obesity treatment.

### Direct body-composition analysis

Table 3 summarizes the body-composition data for all rat groups as measured directly by the dissection method: FM, FFM, FM/FFM, water content (WC), and water percentage (WP). Even though the direct body-composition measurements of all rat groups were significantly lower than those determined by the indirect BIA technique in Table 2, the differences between groups exhibited similar patterns. This was primarily due to human manual errors during dissection, allowing for the determination of FM using only the visceral and subcutaneous fat in the abdominal cavity from animal carcasses. As a result, the FM/FFM ratios were not relevant to the investigation. In comparison to the negative control and all treated rat groups, G-II rats exhibited significantly higher (*p* < 0.05) average FM, FFM, and FM/FFM values, but lower WC and WP values. Although high- and low-dose AuNPs and orlistat treatments significantly reduced FM and FM/FMM and increased WC and WP, there was no statistically significant difference between the four groups, especially for FM and FM/FFM. Nevertheless, as far as we know, these findings have not been reported in any prior scientific study evaluating the body-composition of rats in relation to obesity and its treatment.

**Table 3:** Direct body-composition analysis using the direct dissection method for all study groups of rats.

	FM (g)	FFM (g)	FM/FFM	WC (g)	WP (%)
Negative controls (G-I)	5.75 ± 0.52^bcdef^	112.83 ± 0.58^bcdef^	0.05 ± 0.09^bcdef^	85.47 ± 0.32^bcdef^	75.75 ± 1.50^bcdef^
Positive controls (G-II)	26.11 ± 0.59^acdef^	149.11 ± 0.55^acdef^	0.18 ± 0.07^acdef^	78.94 ± 0.69^acdef^	52.94 ± 1.25^acdef^
Accumulated high-dose (G-III)	12.88 ± 0.61^ab^	131.96 ± 0.69^abef^	0.10 ± 0.08^ab^	83.05 ± 0.37^abef^	62.94 ± 2.00^abef^
Weekly high-dose (G-IV)	13.20 ± 0.39^ab^	131.19 ± 0.46^abef^	0.10 ± 0.08^ab^	82.97 ± 0.17^abef^	63.25 ± 1.45^abef^
Weekly low-dose (G-V)	12.84 ± 0.48^ab^	140.07 ± 1.32^abcd^	0.09 ± 0.6^ab^	80.5 ± 0.52^abcd^	57.47 ± 1.15^abcd^
Orlistat (G-VI)	12.93 ± 0.37^ab^	140.98 ± 1.30^abcd^	0.09 ± 0.08^ab^	80.77 ± 0.60^abcd^	57.29 ± 1.50^abcd^

FM: Fat mass; FP: Fat percentage; FFM: Fat-free mass; WC: Water content; WP: Water percentage (i.e., WC/FFM).

Values are presented as mean ± SD. *P*-value $\:<$ 0.05 against: ^a^G-I, ^b^G-II, ^c^G-III, ^d^G-IV, ^e^G-V, and ^f^G-VI.

### Blood biochemical analysis

**Fig. 4:**
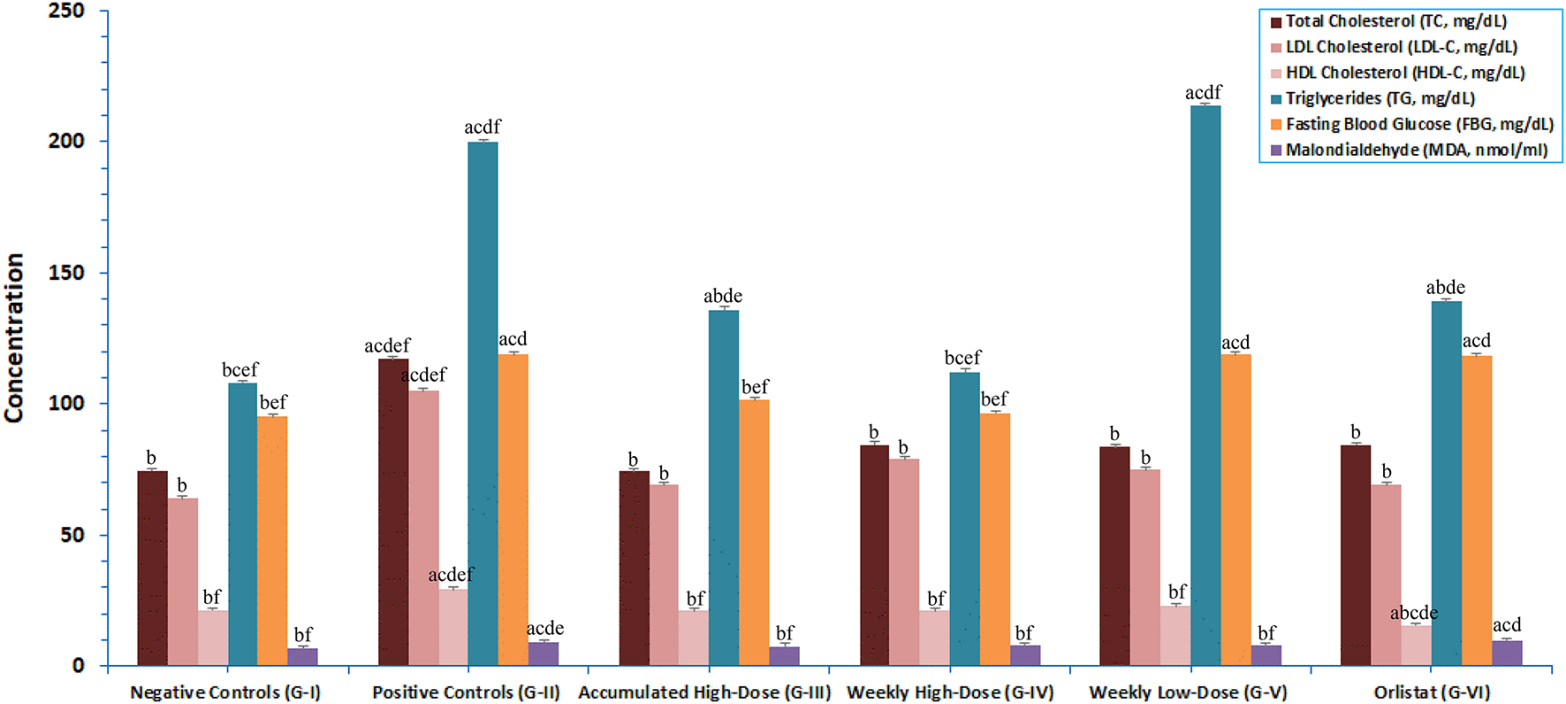
Bar chart plot showing serum concentrations of total cholesterol (TC), low-density lipoprotein cholesterol (LDL-C), high-density lipoprotein cholesterol (HDL-C), triglycerides (TG), fasting blood glucose (FBG), and malondialdehyde (MDA) for HFD-induced obese Wistar Albino rat negative controls (G-I), positive controls (G-II), AuNPs-treated with accumulated high-dose (G-III), weekly high-dose (G-IV), weekly low-dose (G-V), and with orlistat (G-VI). Data are presented as mean ± SD. *P*-value < 0.05 against: ^a^G-I, ^b^G-II, ^c^G-III, ^d^G-IV, ^e^G-V, and ^f^G-VI.

After nine weeks on HFD, the serum levels of TC, LDL-C, HDL-C, TG, FBG, and MDA were significantly (*p* < 0.05) higher in the positive control obese rats G-II compared to the negative control rats G-I on a normal diet, as shown in Fig. 4. High and low doses of AuNPs and orlistat treatments significantly (*p* < 0.5) reduced all markers, except for TG in the low-dose AuNPs G-V, as compared to the positive control obese rats G-II. Moreover, treatment with AuNPs at any dose significantly (*p* < 0.05) lowered rats’ lipid profiles, TG, FBG, and MDA to levels similar to those in negative control rats G-I, in comparison to orlistat.

Previous studies have shown that a single *i.p.* injection of AuNPs at either a low-dose of 0.79 or a high-dose of 7.85 µg/g could significantly lower FM and blood lipid levels in mice, as well as suppress the generation of TNF-α and other cytokines by adipose tissue macrophages^9,10^. FM, which accounts for up to 60% of the weight variation in obese patients, stores over 50% of body TC, with adiposopathic dyslipidemia often associated with increased adiposity, characterized by elevated TG, LDL-C, and HDL-C levels^67^. Moreover, a nine-week therapy with AuNPs decreased glucose intolerance and hyperlipidemia in HFD-obese mice by improving their metabolic and local pro-inflammatory cytokine profiles^9,68^. Furthermore, administration of green synthesized AuNPs at a dose of 1 mg/kg reduced rats’ serum TC and TG levels without changing LDL-C levels^69^. However, oral AuNPs of 2.5 µg/kg for 8 weeks did not affect TC levels in hypercholesterolemic and normal diet-fed rats^70^. Although orlistat promoted weight reduction by blocking fat absorption, it was not the safest choice for obesity management in HFD-obese mice because it increased their food intake, which in turn raised water intake, serum and liver TC and TG levels, and decreased glucose tolerance^55^.

Interestingly, the study showed that treating HFD-obese rats with AuNPs can effectively improve glycemia and obesity-related oxidative damage, restoring FBG and MDA to normal levels. Glycemic control of diabetic rats by AuNPs treatments of variable duration and administration root had been shown to significantly reduce serum FBG levels^71,72^, while mice receiving orlistat showed no improvement or significantly higher serum FBG levels^54,55^. In this study, orlistat-treated rats at a dose of 12 mg/kg had significantly higher serum MDA levels than negative control rats G-I and high-dose AuNPs-treated rats G-III and GIV. In line with this, an earlier study showed that the same dosage of orlistat had no effect on MDA levels in HFD-induced obese rats^54^, while a recent one showed that 10 mg/kg for six weeks reduced oxidative stress damage^73^.

Evidence of the anti-inflammatory and antioxidant effects of AuNPs therapy of 2.5 mg/kg have been shown in Mdx mice with muscular dystrophy and diabetic rats, leading to significantly lower pro-inflammatory cytokines and higher MDA levels^27,71^. By lowering levels of TNF-α and other pro-inflammatory cytokines, AuNPs can suppress inflammation in adipose tissue, which in turn improves metabolic profiles and lessens obesity-related complications like insulin resistance^9,10,12^. They also enhance lipid metabolism and glucose tolerance that causes hyperlipidemia improvement, glucose tolerance normalization, and body weight reduction in HFD-fed mice^55^. It has been shown that the AuNPs processes of angiogenesis inhibition, which converts white to brown adipose tissues, and photothermal lipolysis stimulation can reduce fat storage while promoting energy expenditure^36^. Au@Pt nanozyme has recently shown promise in the treatment of metabolic disorders due to its ability to regulate glucose and lipid metabolism by modulating the expression of several important hepatic genes and gut microbiota profiles^72^.

### Histopathological analysis

**Fig. 5:**
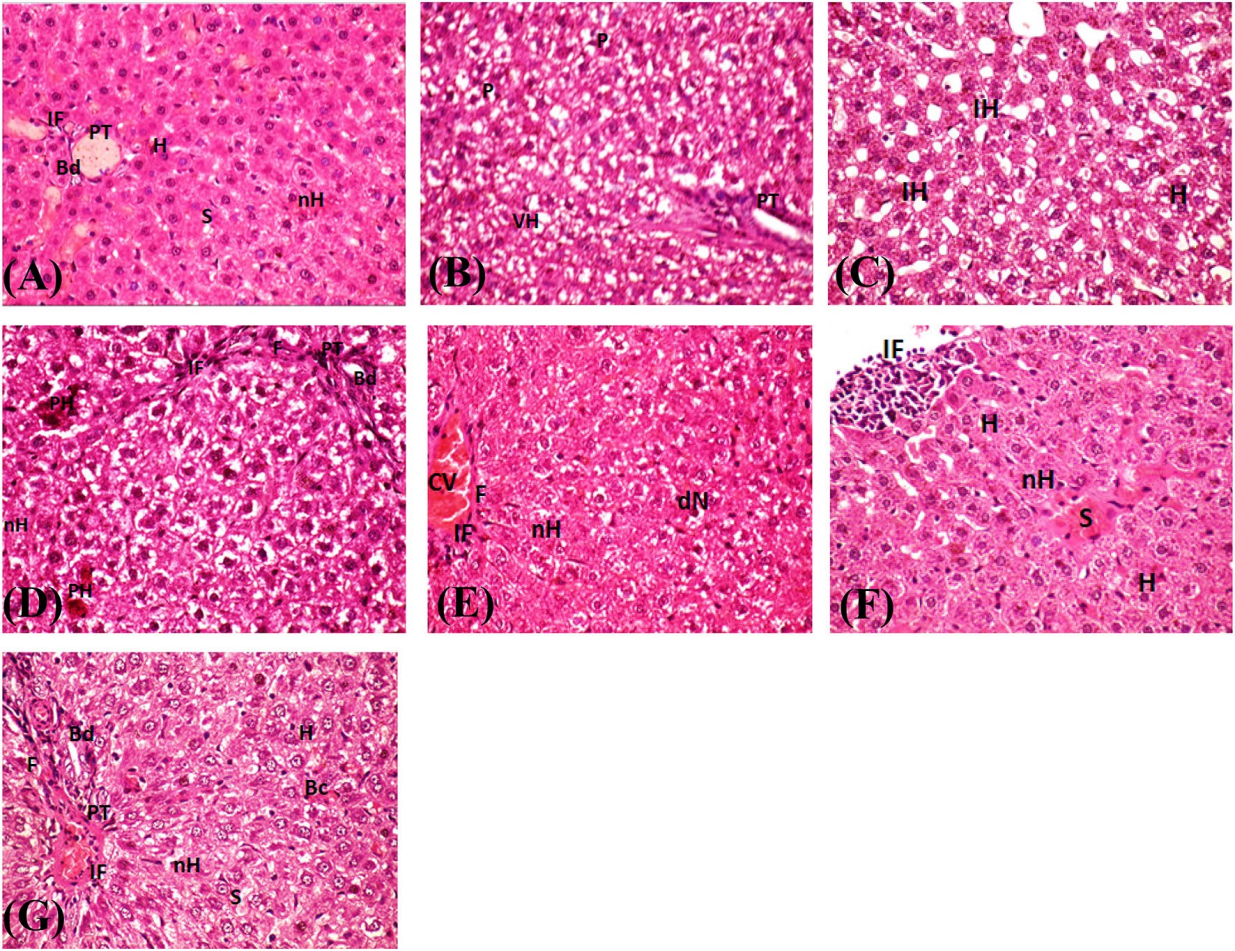
Light microscope photographs of histopathological examination of liver tissues stained with H&E at a magnification of ×400 for HFD-induced obese Wistar Albino rats. Negative controls G-I show portal tract congestion and dilated liver cells (**A**), while positive controls G-II show a dilated portal tract and vacuolated cytoplasm, and hepatocyte steatosis with numerous Ito cells (**B**,**C**). AuNPs accumulated high-dose G-III show few eosinophilic cells, minor cell necrosis, and few vacuolated cytoplasm (steatosis) (**D**). AuNPs weekly high-dose G-IV had disorganized hepatic cells with few vacuolated cytoplasm (steatosis), necrosis cells, and dark nuclei are found. Inflammation is found in the congested central vein region (**E**). AuNPs weekly low-dose G-V show lymphocyte infiltration, radiating cells, a crowded sinusoid, and necrotic hepatocytes (**F**). Orlistat G-VI show proliferating hepatocytes with few necrotic ones, fewer vacuoles in the cytoplasm, congested blood capillaries between hepatic cells, and narrow sinusoids (**G**). Bc: blood capillaries, Bd: bile duct, CV: central vein, dN: dark nucleus, F: fibrotic cells, H: hepatocytes, IF: infiltrating lymphocytes, IH: Ito cells, nH: necrotic hepatocytes, P: pyknotic nuclei, PH: eosinophilic cells, S: sinusoid, PT: portal tract, VH: vacuolated cytoplasm.

**Fig. 6:**
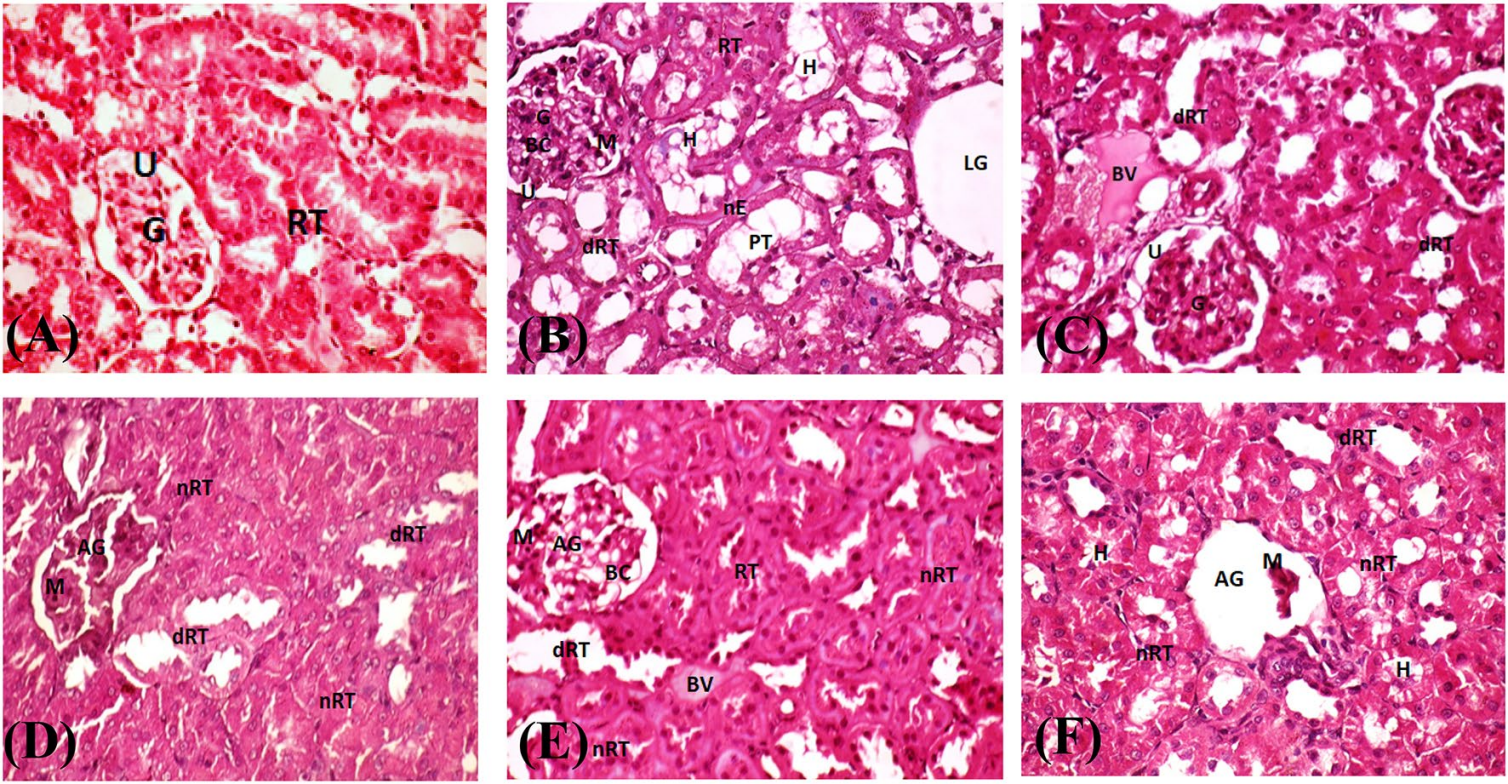
Light microscope photographs of histopathological examination of renal tissues stained with H&E at a magnification of ×400 for HFD-induced obese Wistar Albino rats. Negative controls G-I showed normal glomerulus and well-structured renal tubules (**A**), while positive controls showed lysed glomerulus and dark-nuclei mesangial cells. Proximal tubules had hyaline casts and dilated, necrotic tubular epithelial cells (**B**). AuNPs accumulated high-dose G-III showed organized glomeruli, tubular cell dilation, blood vessel bleed (**C**). AuNPs weekly high-dose G-IV showed an atrophic glomerulus, renal tubule dilation, and epithelial cell necrosis (**D**). AuNPs weekly low-dose G-V showed moderate glomerulus atrophy, congestive blood vessels, and tubular necrosis and dilation (**E**). Orlistat G-VI showed glomerulus collapse, renal tubule dilation, necrosis, and proximal tubules with hyaline casts (**F**). AG: atrophy glomerulus, BC: blood capillaries, BV: blood vessels, dRT: dilated renal tubules, G: glomerulus, H: hyaline casts, LG: lysis glomerulus, M: mesangial cells, nE: necrotic epithelial cells, nRT: necrotic renal tubules, PT: proximal tubules, RT: renal tubules; U: urinary space.

**Fig. 7:**
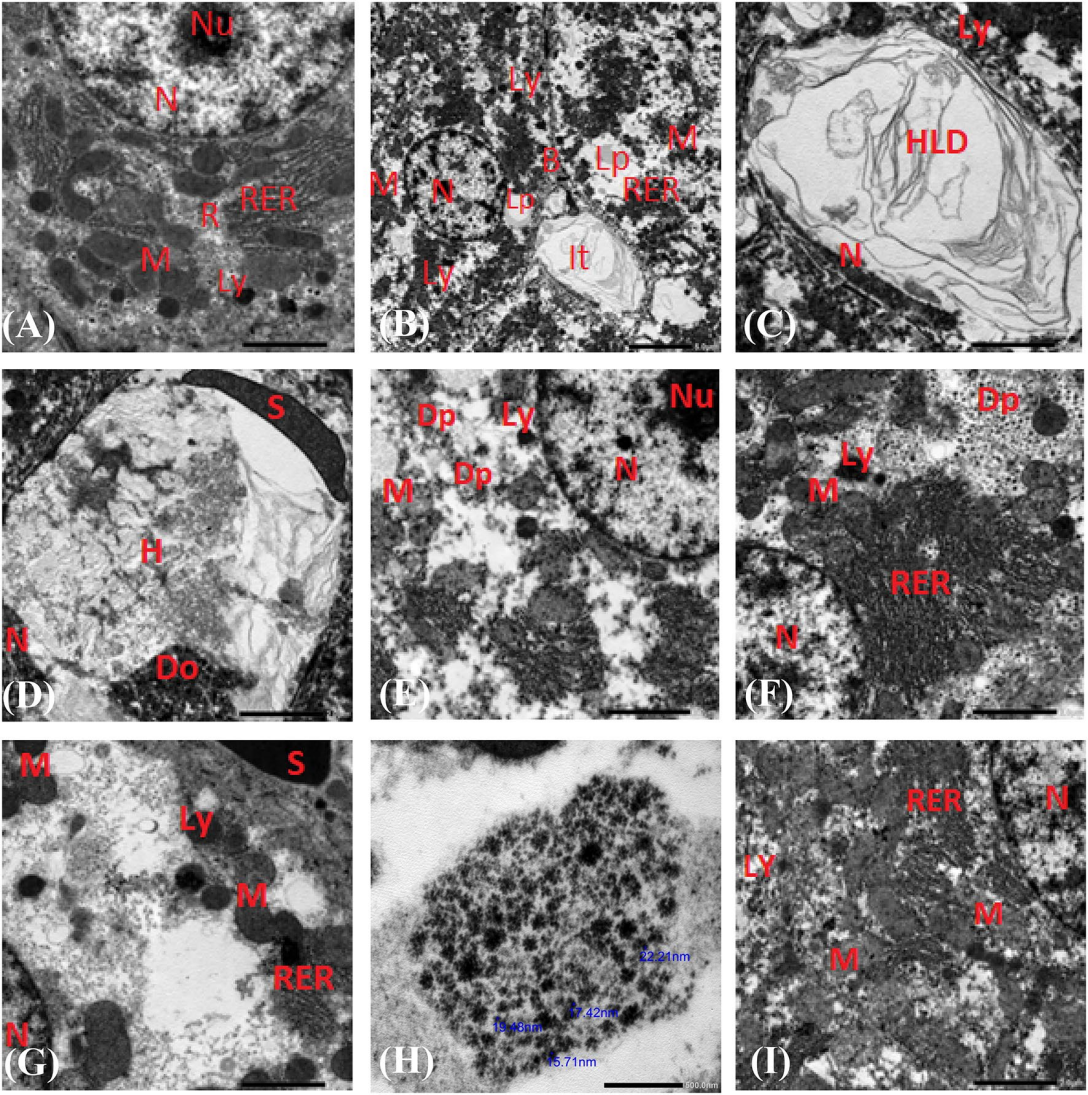
Transmission electron microscopy (TEM) microphotographs of hepatocytes for HFD-induced obese Wistar Albino rats. Negative controls G-I showed a normal-architecture nucleus, mitochondria, and rough endoplasmic reticulum (**A**). Positive controls showed an irregular nuclei-to-cytoplasm ratio, small mitochondria, short rough endoplasmic reticulum cisternae, varied lysosome diameters, large fat droplets, and hyaline lipid casts in Ito cells (B). Spindle-shaped nuclei, undifferentiated organelles, and light, fatty, hyaline-cast cytoplasm, and degenerative organelles are seen were seen (**C**,**D**). AuNPs accumulated high-dose G-III showed a well-organized nucleus, nucleolus, dark body mitochondria, and lysosomes-containing AuNPs (**E**). AuNPs weekly high-dose G-IV showed bright cytoplasm with AuNPs, mitochondria of various sizes and shapes, abundant rough endoplasmic reticulum, and large lysosomes (**F**). AuNPs weekly low-dose G-V showed vacuolated cytoplasm with dark mitochondria, rough endoplasmic reticulum, numerous lysosomes, a congested sinusoid, and AuNPs foci in the vacuolated cytoplasm (**G**,**H**). Orlistat G-VI showed mitochondrial recovery, organized rough endoplasmic reticulum, and absence of lipid droplets (**I**). Scale bar in all micrographs = 2.0 μm, except for B = 5.0 μm, and H = 0.5 μm. B: bile canalicular, Do: depress of the organelles, Dp: dark dots of AuNPs, H: hyaline casts, HLD: hyaline casts of lipid droplet, It: Ito cell, Lp: lipid droplet, Ly: lysosome, M: mitochondria, N: nucleus, Nu: nucleolus, R: ribosomes, RER: rough endoplasmic reticulum; S: sinusoid.

**Fig. 8:**
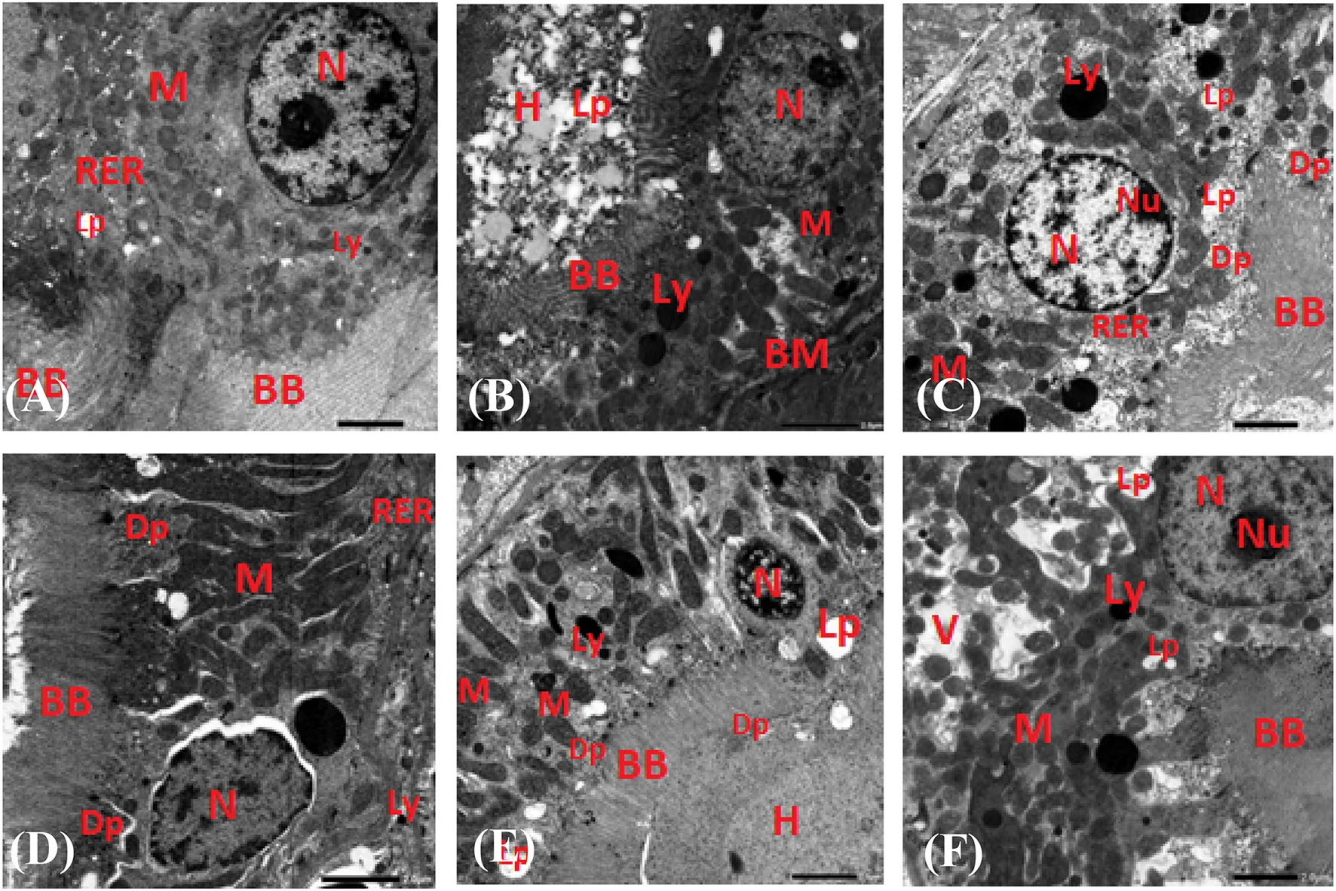
Transmission electron microscopy (TEM) microphotographs of the proximal tubules for HFD-induced obese Wistar Albino rats. Negative controls G-I showed a nucleus with differentiated chromatin, normal mitochondria, and rough endoplasmic reticulum (**A**). Positive controls G-II showed dark cytoplasm, small mitochondria, short rough endoplasmic reticulum, and many-sized lysosomes (**B**). AuNPs accumulated high-dose G-III an organized nucleus with an eccentric nucleolus, restored mitochondria, few lipid droplets, and a regular brush border with AuNPs deposits across its membrane (**C**). AuNPs weekly high-dose G-IV showed an irregular nucleus, restored mitochondria, rough endoplasmic reticulum, an elongated brush border containing precipitated AuNPs, and lysosomes of different sizes (**D**). AuNPs weekly low-dose G-V showed a minuscule nucleus with fragmented chromatin, large fat drops, a dilated brush border, and a dark lumen with hyaline casts (**E**). Orlistat G-VI showed a well-structured nucleus and nucleolus, multiple mitochondria and lysosomes, an irregular brush border, and vacuolated cytoplasm with numerous fat droplets (**F**). Scale bar in all micrographs = 2.0 μm. BB: brush border, BM: basement membrane, Dp: dark dots of AuNPs, H: hyaline casts, Lp: lipid droplet, Ly: lysosomes, M: mitochondria, RER: rough endoplasmic reticulum, N: nucleus, Nu: nucleolus, V: vacuolated cytoplasm.

Light and TEM photographs of the histological examination of stained liver and kidney tissues for Wistar Albino rats are shown in Figs. 5, 6, 7 and 8. Hepatic tissues of positive controls G-II showed severe steatosis, many Ito cells, and organelle abnormalities due to fat accumulation in hepatocytes after nine weeks of HFD-induced obesity (Figs. 5B and C and 7B). AuNPs or orlistat-treated rats had hepatic structural alterations compared to the negative control group G-I, but less evidence of steatosis and cell necrosis compared to the positive control group G-II (Fig. 5D and F). While fibrotic cells and lymphocytes were present in all treated rats’ hepatic tissues, lymphocytic infiltrations in large foci were markedly increased in hepatic tissues treated with low doses of AuNP (Fig. 5F), together with fat vacuoles filling the cytoplasm (Fig. 7G and H). Treatment with high doses of AuNPs (G-III and G-IV) reduced hepatocyte fat area while simultaneously restoring cell organelles and accumulating AuNPs in the cytoplasm and mitochondria (Fig. 7E and F). Lopez-Chaves et al.,^74^ hypothesized that nanoparticles like AuNPs are chemically or physiologically digested before being deposited in fat droplets to decrease hepatocyte toxicity. Evidence suggests that the histological changes in rat hepatic tissues are dose-dependent; with blood vessel congestion at 25 mg/kg AuNPs, lymphocyte infiltration at 100 mg/kg, and hepatocytes necrosis at 250 mg/kg^75^. Hepatocytes proliferation, with modest amounts of necrosis and fibrotic lesions (Fig. 5G), in addition to mitochondria and rough endoplasmic reticulum restoration and the elimination of fat droplets was the overall result of orlistat treatment (Fig. 7I). As expected, daily orlistat at 10 mg/kg reduced hepatic inflammation and fat deposits but did not entirely restore them^2^. Conversely, orlistat induced severe hepatic damage and ultrastructure alterations in albino rat liver cells^76^.

Renal tissues of negative control rats G-I had normal glomerular and tubular structures (Figs. 6A and 8A) but positive control rats G-II had fatty tissues, glomerular lysis, tubular epithelial cell dilation and necrosis, and hyaline casts in the proximal tubules (Figs. 6B and 8B). When compared to G-II, high doses of AuNPs improved glomerular structure and reduced tubular dilation, although renal tubules did not fully recover. In addition, the presence of fatty tubules could be reduced by treatment with AuNPs, irrespective of the dosage (Figs. 6C to F and 8C to F). However, a few dilated and packed tubules and hemorrhage in the glomerular tubules blood vessels were still seen in the AuNPs high-dose G-III renal tissues (Fig. 6C). These observations are supported by the findings that AuNPs treatments were associated with minor glomerular and tubular atrophy, indicating size-dependent renal tissue histological alterations in rats^75,77^. At 250 mg/kg, AuNPs has been shown to induce congestion of blood vessels, destruction of renal tubules, and necrosis in rats’ renal tissues^75^. Furthermore, renal tissues of AuNPs weekly high- and low-dose treated rats (G-IV and G-V) showed atrophic glomeruli and tubular cell necrosis, although high doses reduced fat cells and repaired renal cell structure better than low doses (Figs. 6C to E and 8C and E). AuNPs has been shown to improve the renal cell histopathological modification due to their anti-inflammatory and antioxidant effects^78^. It is worth noting that orlistat-treated G-VI renal tissues had glomerulus collapse, renal tubule dilation, necrosis, and proximal tubules filled with hyaline casts (Fig. 6F). Besides, the proximal tubules showed a cell with cytoplasm loaded with lipid vacuoles and an irregular structure except for the organized nucleus with nucleolus (Fig. 8F). Therefore, AuNPs are a potential alternative to orlistat for treating obesity because they are more tolerable, have fewer side effects, and are more effective.

## Conclusion

In the Wistar Albino rat model of obesity, BIA has proven to be a sensitive method for calculating body-composition, distinguishing between FM and FFM, and offering useful insights into the FM/FFM ratio to measure health status, unlike direct evaluation. BIA and dissection measurements of body-composition showed that high doses of AuNPs treatment significantly lowered body FM and improved FFM, TBW, and consequently muscle masses. Thus, BIA is better than other methods of assessing body-composition since it is safe, fast, and inexpensive, making it an ideal choice for studies and trials that need repeated measurements. Moreover, giving rats daily (7.48 µg/kg) or weekly (52.33 µg/kg) high-dose AuNPs effectively controlled dyslipidemia, glycemia, and antioxidant effects. They restored normal lipid profiles, TG, FBG, and MDA levels by reducing obesity-related oxidative damage. Notably, histological and ultrastructural examinations showed that daily administration of high-dose AuNPs restored liver and kidney cells, and decreased fat accumulation compared to orlistat, which is the standard treatment for obesity. However, more research is required to decide whether these particles pose a risk to human organs.

## Data Availability

Metadata used and/or analyzed during the current study will be made available from the corresponding author on reasonable request.
